# Impact of the COVID-19 pandemic on endometrial cancer diagnosis: a retrospective observational study

**DOI:** 10.61622/rbgo/2026rbgo350

**Published:** 2026-05-12

**Authors:** Júlia Simioni Freitas, Júlio Cesar Teixeira, Cristina Laguna Benetti-Pinto, Daniela Angerame Yela

**Affiliations:** 1 Universidade Estadual de Campinas Campinas SP Brazil Universidade Estadual de Campinas, Campinas, SP, Brazil.

**Keywords:** Endometrial neoplasms, Hysteroscopy, COVID-19, Pandemics, Postemenopause, Uterine hemorrhage

## Abstract

**Objective:**

To compare the prevalence of diagnostic hysteroscopies for endometrial cancer, the prevalence of endometrial cancer diagnoses, and the staging of endometrial cancer at the time of diagnosis before and during the COVID-19 pandemic.

**Methods:**

Retrospective observational study included 631 women who underwent diagnostic hysteroscopy at a tertiary hospital in Campinas. Medical records were analyzed from two periods: two years before the COVID-19 pandemic (2018 and 2019) and two years during the pandemic (2020 and 2021).

**Results:**

The frequency of hysteroscopies before the pandemic was 59.9%, compared to 40.1% during the pandemic. There were no significant differences in average age, body mass index, or cancer prevalence between the two periods. Among women who underwent hysteroscopy during the pandemic, a lower prevalence of comorbidities and postmenopausal status was observed (p = 0.029 and p = 0.004, respectively). During this period, the primary indication for hysteroscopy was abnormal uterine bleeding (p = 0.004).

**Conclusion:**

The pandemic led to a reduction in the number of women with comorbidities undergoing hysteroscopy and an increase in indications due to abnormal bleeding. This scenario may suggest that women at higher risk of COVID-19 complications were less likely to seek medical care.

## Introduction

Endometrial cancer is one of the most common malignancies of the female genital tract worldwide, with more than 417,000 diagnoses in 2020, ranking sixth among cancers in women.^(1)^ In Brazil, it is the seventh most common type of cancer in women, with an estimated 7,840 new cases expected to be diagnosed in 2023-2025, according to the Brazilian National Cancer Institute (INCA).^(2)^

The main symptom is abnormal uterine bleeding (AUB), which occurs in 75% to 90% of women diagnosed with endometrial cancer.^(3)^ Hysteroscopy with biopsy is considered the gold standard for diagnosing intrauterine lesions.^(4)^ Abnormal uterine bleeding is a common symptom of endometrial cancer and a source of considerable distress for women. Endometrial biopsy, especially when combined with outpatient hysteroscopy, is effective for diagnosing cancer. This allows for early detection and higher survival rates.^(5)^

However, due to the COVID-19 pandemic that began in 2020, elective appointments and routine exams were canceled, and many women avoided seeking medical care—even when experiencing symptoms—due to fear of infection by the SARS-CoV-2 virus. As a result, fewer cases of endometrial cancer may have been diagnosed, or they may have been diagnosed at more advanced stages, which would hinder early detection and treatment.^(6)^

Therefore, this study aimed to compare the prevalence of hysteroscopy exams for diagnosing endometrial cancer, the prevalence of endometrial cancer diagnoses, and the staging of endometrial cancer at the time of diagnosis before and during the COVID-19 pandemic.

## Methods

A retrospective observational study was conducted with women who underwent hysteroscopy at a tertiary hospital in Campinas between 2018 and 2021.

The hospital where the study was conducted is one of the only public hospitals that performs hysteroscopy. It serves not only the city of Campinas, which has over 1 million inhabitants, but also other cities such as Americana, Artur Nogueira, Cosmópolis, Holambra, Hortolândia, Indaiatuba, Itatiba, Jaguariúna, Monte Mor, Morungaba, Nova Odessa, Paulínia, Pedreira, Santa Bárbara d’Oeste, Sumaré, Valinhos, Vinhedo, Águas de Lindóia, Amparo, Lindóia, Monte Alegre do Sul, and Serra Negra.

Participants were divided into two groups based on the year the hysteroscopy was performed: before the COVID-19 pandemic (procedures performed from January 2018 to December 2019) and during the pandemic (procedures performed from March 2020 to December 2021).

All women who underwent hysteroscopy during the study period were eligible for inclusion. Exclusion criteria included missing data from medical records, inability to perform outpatient hysteroscopy due to pain or cervical stenosis, and cases in which the procedure was solely for intrauterine device insertion or removal.

The following variables were extracted from the medical records: age, ethnicity (categorized as white or non-white), parity, Body Mass Index [BMI was categorized as: underweight (<20 kg/m²), normal weight (20–25 kg/m²), overweight (25–30 kg/m²), and obesity (> 30 kg/m²)], presence of comorbidities (including hypertension, diabetes mellitus, or other conditions), smoking status, time since menopause (in years), presence of abnormal uterine bleeding, indication for hysteroscopy (abnormal bleeding or asymptomatic - endometrial thickening), use of hormonal therapy (estroprogestin or tibolone), histopathological diagnosis (classified as benign—e.g., myoma, endometrial polyp, hyperplasia without atypia—or malignant—e.g., endometrial cancer or hyperplasia with atypia), and staging of endometrial cancer.

Cancer staging was based on the 2014 FIGO classification: Stage I (tumor confined to the uterus), Stage II (tumor invades cervical stroma), Stage III (local and/or regional spread), and Stage IV (invasion of the bladder and/or bowel mucosa and/or distant metastasis).^(7)^

To describe the sample profile according to the study variables, frequency tables were constructed for categorical variables, presenting absolute frequencies and percentages. For numerical variables, descriptive statistics were used, including mean, standard deviation, minimum and maximum values, median, and quartiles.

To compare categorical variables, Fisher's Exact Test or the Chi-square test was applied. For continuous variables, the Mann-Whitney test was used. The Cochran-Armitage test was employed to evaluate temporal trends. The effect size was determined using the Coehn test, where values from 0.20 to 0.49 showed a small effect size, values from 0.50 to 0.79 a medium effect size, and values greater than 0.80 a large effect size.

A significance level of 5% was adopted for all statistical tests. Analyses were performed using the SAS System for Windows (Statistical Analysis System), version 9.4.

This study was approved by the institution's Research Ethics Committee number: #4.985.710 (protocol number: 58583122.5.0000.5404).

## Results

Out of 700 women initially eligible. Of these, 69 women were excluded (42 did not undergo hysteroscopy due to pain or cervical stenosis, 11 underwent hysteroscopy to remove an IUD, and 16 because there was insufficient data in their medical records), and 631 women medical records were analyzed.

The mean age of the women was 54.4±11.9 years, and the average time since menopause was 12.9±8.4 years. The annual distribution of hysteroscopies was as follows: 27.42% in 2018, 32.49% in 2019, 18.07% in 2020, and 22.03% in 2021. Most of the women were postmenopausal (67.0%), and the overall prevalence of endometrial cancer was 8.8% (Table 1).

**Table 1:** Sociodemographic and clinical characteristics of women undergoing hysteroscopy

Variable	Mean±SD	n(%)
Age (years)	54.4±11.9	
White ethnicity		501(81.6)
Nulligravida		69(12.5)
Smoking		53(8.4)
Time since menopause (years)	12.9±8.4	
Year of hysteroscopy		
2018		173(27.4)
2019		205(32.5)
2020		114(18.1)
2021		139(22.0)
Comorbidities		328(54.3)
Postmenopausal		398(67.0)
Abnormal uterine bleeding		404(64.0)
Endometrial câncer		56(8.8)

SD = standard deviation

A comparison of the pre-pandemic and pandemic periods revealed no significant sociodemographic differences between the groups. However, during the pandemic, the women who underwent hysteroscopy had fewer comorbidities (p=0.029), were less frequently postmenopausal (p=0.004), and most commonly presented with abnormal uterine bleeding (p=0.004). There were no significant differences in the prevalence of endometrial cancer diagnosed via hysteroscopy or in cancer staging between the two periods (Table 2).

**Table 2:** Characteristics before and during the COVID-19 pandemic

Variable		Before Mean±SD/n(%) (n=378)	During Mean±SD/n(%) (n=253)	p-value	Cohen
Age (years)		55.0±11.7	53.5±12.1	0.120 *	0.13
Body mass index (kg/m²)		30.4±7.0	30.6±6.1	0.501 *	0.03
Number of pregnancies		2.7±2.0	2.7±2.0	0.959 *	0.00
Comorbidities (yes)		207(57.9)	121(48.9)	0.029 **	0.18
Postmenopausal (yes)		252(71.5)	146(60.3)	0.004 **	0.24
Abnormal uterine bleeding		225(59.5)	179(70.7)	0.004 **	0.24
Indication				0.023 **	
Abnormal uterine bleeding		206(54.5)	165(65.2)		0.22
Endometrial thickening		117(30.9)	53(20.9)		0.23
Other		55(14.5)	35(13.8)		0.02
Endometrial cancer (yes)		35(9.2)	21(8.3)	0.678 **	0.03
Staging				0.908 ***	
Hyperplasia with atypia		11(34.3)	6(28.5)		0.13
	Stage I	6(18.7)	4(19.0)		0.01
	Stage II	10(31.2)	6(28.5)		0.06
	Stage III	3(9.3)	4(19.0)		0.28
	Stage IV	2(6.2)	1(4.7)		0.07

SD = standard deviation

* Mann-Whitney Test;

** Chi-square Test;

*** Fisher's Exact Test

When analyzing data across the four years, we observed a significant decline in comorbidities such as hypertension and diabetes mellitus, particularly in 2021. Clinically, there was an increasing trend in the indication for hysteroscopy in women presenting with abnormal uterine bleeding, while indications based on asymptomatic findings (e.g., endometrial thickening) declined (Table 3).

**Table 3:** Annual trend analysis (2018–2021)

Variable	2018 n(%) (n=173)	2019 n(%) (n=205)	2020 n(%) (n=114)	2021 n(%) (n=139)	p-value *
Endometrial cancer (yes)	13(7.3)	22(10.7)	10(8.7)	11(7.9)	0.956
Systemic arterial hypertension	86(53.4)	96(50.5)	51(45.1)	53(39.8)	0.015
Diabetes mellitus	44(27.3)	44(22.6)	26(23.0)	19(14.2)	0.012
Postmenopausal (yes)	121(74.6)	131(68.9)	72(65.4)	74(56.0)	<0.001
Abnormal uterine bleeding (yes)	96(55.4)	129(62.9)	76(66.6)	103(74.1)	<0.001
Indication – Abnornal uterine bleeding	89(51.4)	117(57.0)	71(62.2)	94(67.6)	0.003
Indication – Endometrial thickening	56(32.3)	61(29.7)	29(25.4)	24(17.2)	0.002

* Cochran-Armitage test

## Discussion

Before the pandemic, 378 hysteroscopies were performed (59.9%) and during the pandemic, 253 procedures (40.1%). The overall prevalence of endometrial cancer was 8.8%. No significant differences were found in mean age, body mass index, or cancer prevalence between the two periods. However, during the pandemic, it was less frequent women with comorbidities and it was less frequent postmenopausal women underwent hysteroscopy. Additionally, most procedures during this period were indicated for abnormal uterine bleeding. There were no differences in the prevalence or staging of endometrial cancer between the pre-pandemic and pandemic periods.

A reduction in the number of diagnostic hysteroscopies performed during the COVID-19 pandemic was observed. This finding aligns with other studies that reported a general decline in cancer screening, diagnosis, treatment, and supportive care during the pandemic.^(8,9)^

A Spanish study involving gynecologic oncologists reported that the COVID-19 pandemic significantly affected their clinical routines, with over half indicating a decline in the diagnostic and therapeutic quality of care. Common measures implemented included increased use of telemedicine, restrictions on outpatient visits to high-risk or symptomatic patients, and mandatory SARS-CoV-2 screening before surgery using nasopharyngeal swab PCR. Most respondents reported no changes in surgical approaches or treatments for ovarian, endometrial, or cervical cancers during the pandemic.^(10)^ These findings are consistent with our results, suggesting that women at higher risk of complications from COVID-19 (e.g., older women and those with comorbidities) were less likely to seek hospital-based care.

A Japanese cohort study evaluating gynecologic cancer prevalence from 2018 to 2021 found no change in the prevalence of endometrial cancer and staging of endometrial cancer before and during the pandemic.^(11)^ These results were similar to our study. That study also observed no difference in the prevalence and staging of ovarian cancer, but observed an increase in advanced-stage cervical cancer during the pandemic.^(11)^ A Dutch population-based study also reported no changes in endometrial cancer diagnoses during the pandemic between 2017 and 2020.^(12)^

An Italian single-center study found that most endometrial cancer diagnoses were made based on symptoms, and no delays in diagnosis or changes in cancer staging were observed when compared to the pre-pandemic period.^(13)^ An American study has shown diagnoses of endometrial cancer in more advanced stages during the pandemic.^(14)^

These findings suggest that the stable prevalence of endometrial cancer may be due to the nature of its primary symptom—abnormal uterine bleeding—which is both early and distressing, prompting women to seek medical attention. A literature review on endometrial cancer diagnosis in asymptomatic women showed that such cases were less likely to present in advanced stages.^(15)^

In an American study of endometrial cancer during the beginning of the COVID-19 pandemic, endometrial cancer incidence rates decreased, but the proportion of metastatic cancer increased.^(16)^

The COVID-19 pandemic has impacted healthcare systems, leading to a temporary decline in cancer screenings, diagnostics, and elective medical procedures.^(17)^ With the rapid spread of SARS-CoV-2 in early 2020, healthcare systems prioritized pandemic response efforts, recommending the suspension of non-essential medical appointments and elective surgeries to conserve healthcare resources and reduce viral transmission. As a result, routine gynecological evaluations, diagnostic imaging, and biopsies were postponed, directly impacting endometrial cancer detection rates.^(18)^ Fear of potential exposure to the virus in healthcare settings also discouraged many people from seeking routine medical care, exacerbating the decline in cancer screenings.^(17)^ Unlike other malignancies, endometrial cancer often presents with noticeable symptoms, such as abnormal uterine bleeding. This symptom often prompts individuals to seek medical care quickly, increasing the chances of early detection.^(16)^

This study is limited by its retrospective design, use of medical record data, and single-center setting, which may not reflect broader trends across Brazil. Another important limitation is the exclusion of medical records with missing data. The study's strengths lie in its use of primary data and in being one of the few Brazilian studies that assessed the prevalence of endometrial cancer during the pandemic. During the pandemic, fewer hysteroscopy exams were performed. We infer that this reduction stemmed from women's fear of contracting the COVID-19 virus. We also observed a reduction in the number of women with more serious illnesses and in older women. We believe this is also due to fear of the pandemic. We believe that there was no reduction in the prevalence of endometrial cancer because women with uterine bleeding continued to seek the service and this is the main symptom of endometrial cancer and that is why this may have helped maintain the number of endometrial cancer diagnoses.

## Conclusion

The COVID-19 pandemic may have contributed to a reduction in the number of diagnostic hysteroscopies performed. It also led to a decrease in the number of women with comorbidities undergoing the procedure and an increase in the proportion of hysteroscopies performed due to abnormal uterine bleeding. This scenario suggests that women at increased risk of COVID-19 complications were less likely to seek medical attention.

## Data Availability

The research data are described in the article presented.
